# Supplementary material for: Identification of A Novel Arsenic Resistance Transposon Nested in A Mercury Resistance Transposon of Bacillus sp. MB24
Source: Microorganisms. 2019 Nov 16;7(11):566. doi: 10.3390/microorganisms7110566 (PMC6920998; doi:10.3390/microorganisms7110566)
Supplement: Supplementary file 1 [file microorganisms-07-00566-s001.pdf]

Table S1. Primers used in this study.

Primer	Sequence (5′-3′)	Application
For-arsR1	CGAATTGTGTCAGCGTGGAG	5′-RACE of *arsR1*
Rev-arsR1	GGTTTGGGTAGTTGATTGAGTGT	5′-RACE of *arsR1*
For-ORF3	GGTTTGGGTAGTTGATTGAGTGT	5′-RACE of orf*3*
Rev-ORF3	CGATTCCCAGCCATGCTGTT	5′-RACE of orf*3*
For-arsR2	ACGGAGTATGCAGCAGGATT	5′-RACE of *arsR2*
Rev-arsR2	TTGGGACGCTCTTGTTACCT	5′-RACE of *arsR2*
RTarsR1-F	ACTCAATCAACTACCCAAACCTT	RT-PCR of *arsR1*
RTarsR1-R	TGCTCAGATAATAGTGCATCTAC	RT-PCR of *arsR1*
RTorf3-F	TAGAACAATACTTACAGCCGCTT	RT-PCR of orf*3*
RTorf3-R	GAGAGATTGTTCTTCTTTCAACC	RT-PCR of orf*3*
RTarsR2-F	AGAGCGTCCCAATTACTGAAACT	RT-PCR of *arsR2*
RTarsR2-R	GTTTGGTCTGGTACATGTGCTAA	RT-PCR of *arsR2*
16S-F	GCCACGAGCCGCGGT	Normalization in RT-PCR
16S-R	ACGGGCGGTGTGTAC	Normalization in RT-PCR

Table S2. Gene analysis of Tn*ARS*1 based on Blastp.

Gene	Amino Acid (length)	Putative Protein	Identity (%)	Similarity (%)	Reference Species (Accession Number)
rec	210	recombinase	97	99	*Bacillus wiedmannii* (PTC10770.1)
arsR1	*101*	transcriptional regulator/Arsenical Resistance Operon Repressor	99	100	*Bacillus cereus* group (PES99194.1)
orf3	*435*	glutamate synthase	99	99	*Bacillus cereus* group (PES99193.1)
orf4	352	flavoprotein monooxygenase	100	100	*Bacillus cereus* group (PES99191.1)
orf5	111	GNAT family N-acetyltransferase	100	100	*Bacillus cereus* group (PES99190.1)
arsR2	115	ArsR family transcriptional regulator	100	100	*Bacillus cereus* group (PES99188.1).
arsB	351	ArsB (arsenite transporter)	100	100	*Bacillus cereus* group (PES99187.1).
arsC	134	ArsC (arsenate reductase)	100	100	*Bacillus cereus* group (PES99186.1).
arsD	119	ArsD (*ars* operon transcriptional repressor)	100	100	*Bacillus cereus* group (PES99185.1).
arsA	586	ArsA (arsenical pump-driving ATPase)	99	99	*Bacillus cereus* group (PES99184.1).
orf11	93	adhesin	100	100	*Bacillus cereus* group (PES99183.1)
orf12	141	protein phosphatase	100	100	*Bacillus cereus* group (PES99182.1)
tnpA	986	transposase	96	98	*Bacillus stratosphericus* (KML06687.1)
